# Auranofin Inhibits Retinal Pigment Epithelium Cell Survival through Reactive Oxygen Species-Dependent Epidermal Growth Factor Receptor/ Mitogen-Activated Protein Kinase Signaling Pathway

**DOI:** 10.1371/journal.pone.0166386

**Published:** 2016-11-15

**Authors:** Xiaodong Chen, Radouil Tzekov, Mingyang Su, Haiyan Hong, Wang Min, Aidong Han, Wensheng Li

**Affiliations:** 1 Xiamen Eye Center of Xiamen University, Xiamen University, Xiamen, Fujian, China; 2 Department of Ophthalmology, First Hospital of Xi'an, Shanxi Institute of Ophthalmology, Shanxi Provincial Key Laboratory of Ophthalmology, Xi'an, Shanxi, China; 3 State Key Laboratory for Cellular Stress Biology, School of Life Sciences, Xiamen University, Xiangan, Xiamen, China; 4 Department of Ophthalmology, University of South Florida, Tampa, Florida, United States of America; 5 The Roskamp Institute, Sarasota, Florida, United States of America; 6 Interdepartmental Program in Vascular Biology and Therapeutics, Department of Pathology, Yale University School of Medicine, New Haven, Connecticut, United States of America; Faculty of Medicine & Health Science, UNITED ARAB EMIRATES

## Abstract

Abnormal survival of retinal pigment epithelium (RPE) cells contributes to the pathogenesis of proliferative vitreoretinopathy (PVR), a sight-threatening disease. In this study, we explored the effect of the anti-rheumatic agent auranofin (AF) on RPE cell survival and studied the underlying signaling mechanisms in vitro. Our results showed that AF inhibited ARPE-19 cell survival in a dose and time-dependent manner. Application of AF induced several effects: a significant decrease in total epidermal growth factor receptor (EGFR) and an increase in phosphorylated EGFR and mitogen-activated protein kinase (MAPK), including extracellular signal-regulated kinase (ERK), P38 mitogen-activated protein kinase (P38MAPK), c-Jun N-terminal kinase (JNK), c-Jun, mitogen activated protein kinase activated protein kinase 2(MAPKAPK2), and heat shock protein 27 (HSP27). AF also inhibited epidermal growth factor (EGF)-dependent cell proliferation and migration through affecting EGFR/MAPK signaling. The antioxidant N-acetylcysteine (NAC) blocked the AF-induced increase of reactive oxygen species (ROS) production, the reduction of total EGFR, and the phosphorylation of multiple nodes in EGFR/MAPK signaling pathway. P38MAPK inhibitor SB203580, but not inhibitors of EGFR (erlotinib), ERK (FR180204) and JNK (SP600125), suppressed AF-induced phosphorylation of EGFR/p38MAPK/MAPKAPK2/Hsp27. In conclusion, the ROS-dependent phosphorylation of EGFR/MAPK is an important signaling pathway for AF-induced inhibition of RPE cell survival, and AF may have the potential for treatment of abnormal survival of RPE cells in PVR.

## Introduction

Proliferative vitreoretinopathy (PVR) is caused by the growth and contraction of cellular membranes within the vitreous cavity and on retinal surfaces, and is a sight-threatening disease [1]. Retinal pigment epithelium (RPE) and glial cells were identified as main participants in the pathophysiology of PVR [2]. RPE cells are located between the neural retina and the choroid and are considered vital for a normal visual function [3]. RPE cell proliferation and migration following retina detachment or trauma have been considered as a key element in the induction of PVR[1]. This process resembles fibrotic wound healing by the RPE cells. Although many efforts have been reported in trying to solve the problem by inhibiting cell proliferation, successful and long-lasting treatment of PVR remains a challenge.

Several lines of evidence reveal that growth factors and cytokines, as major intercellular messengers, are involved in the pathogenesis of PVR [4]. For example, epidermal growth factor receptor (EGFR) is a tyrosine kinase receptor located in the cell membrane, which can bind to the epidermal growth factor (EGF) and can induce various intracellular signal transduction pathways. Such pathways could involve mitogen-activated protein kinase (MAPK) signaling, including P38 mitogen-activated protein kinase (P38MAPK), extracellular signal-regulated kinases (ERK) signaling, and c-Jun N-terminal protein kinase (JNK) signaling. Activation of these pathways could lead to cell proliferation and migration [5]. Several groups have already indicated that EGF enhances RPE cell survival and can stimulate proliferation and migration of RPE cells through activation of the EGFR signaling pathway [6–8]. Some studies show that the activation of EGF seems to be an important factor of the pathogenesis of PVR [9–11].

Auranofin (AF; 2,3,4,6-tetra-O-acetyl-1-thio-β-D-glucopyra-nosato-S-[triethylphosphine] gold) is a gold-containing compound that was initially developed for the treatment of rheumatoid arthritis[12]. Some data suggest that AF has also potential for the treatment of other diseases, such as cancer, neurodegenerative disorders and infectious diseases [13]. Studies have showed that AF inhibited EGF binding to HeLa cells and enhanced protein kinase C-mediated EGFR phosphorylation in epidermoid carcinoma cell line [14,15]. It was also reported that AF can activate different kinases participating in signaling cascades controlling cellular responses to cytokines and stress, like P38MAPK, ERK, and JNK [16,17]. In our previous study, we found that AF inhibited cell survival in endothelial cell lines that were derived from axillary lymph node/vascular epithelium by down-regulating vascular endothelial growth factor receptor-3 and inducing P38MAPK phosphorylation [18]. However, it is unclear whether AF acts also through additional mechanisms, involving EGFR/MAPK signaling in RPE cells.

In this study, we sought to further investigate whether AF affects the survival and proliferation of RPE cells in vitro and focused on the effects of AF on EGF/EGFR/MAPK signaling pathway. Our results reveal that AF can inhibit the survival of RPE cells through reactive oxygen species (ROS)-dependent phosphorylation of EGFR/MAPK signaling pathway. Overall, the data obtained in this and our previous study, suggest that AF has the potential to be used as a therapeutic agent for the treatment of abnormal survival of RPE cells in PVR.

## Materials and Methods

### Materials

Auranofin (AF) was purchased from Abcam Inc. (Cambridge, MA, USA). The 3-(4,5- Dimethyl-2-thiazolyl)-2,5-diphenyl-2H-tetrazolium bromide (MTT), N-acetylcysteine (NAC), dimethylsulfoxide (DMSO) and 2’,7’-dichlorodihydrofluorescein diacetate (DCFDA) were purchased from Sigma-Aldrich (St. Louis, MO,USA). SB203580 was purchased from Santa Cruz Biotechnology (Dallas, TX, USA). Erlotinib, SP600125, FR180204, and Bromodeoxyuridine (BrdU) were purchased from Selleck Chemicals LLC (Houston, TX, USA). Primary antibodies and secondary antibodies were purchased from Cell Signaling Technology (Danvers, MA, USA), except BrdU primary antibody, which was purchased from Proteintech (Wuhan, Hubei, China). Alexa Fluor^®^488- or 594-conjugated secondary antibodies were from Abcam Inc. (Cambridge, MA, USA).

### Cell culture and agents preparation

The human RPE cell line ARPE-19 was purchased from American Type Culture Collection (ATCC, Manassas, VA, USA). Cells were cultured to 80% confluence in Dulbecco's Modified Eagle Medium:Nutrient Mixture F-12 with 10% fetal bovine serum, 100 units/ml Penicillin and 100 μg/ml Streptomycin, at 37°C in a humidified incubator with 5% CO_2_ atmosphere. AF was dissolved in DMSO (≥99.7%) to prepare 0.5 mM, 1.0 mM, 2.0 mM, 3.0 mM AF stock solution and diluted (1:1000) in culture media to administer to treatment group cells. Same volume DMSO was added into control group cells. Erlotinib, SB203580, SP600125, FR180204, and Bromodeoxyuridine were also dissolved in DMSO (≥99.7%) and NAC was dissolved in sterile H_2_O to prepare 1000 mM NAC stock solution and diluted (1:1000) in culture media to administer to treatment group cells.

### Cell viability inhibition assay (MTT assay)

ARPE-19 cells (1×10^4^/well) were cultured in 96-well culture plates at 37°C in 5% CO_2_ humid environment for 12 hours. Cells were then treated with different dose of AF or solvent for 12 hours. 10μL solution of MTT (0.5mg/ml) was added to each well. After incubation for 4 hours, the culture medium was removed and 150μL dimethylsulfoxide was added into each well. The absorbance was detected at 570nm with a microplate reader (POLARstar Omega, BMG Labtech). Cell viability was expressed as a percentage of control.

### Flow cytometric analysis of cell death

ARPE-19 cells (1.0×10^5^/well) in 12-well culture plates were treated with different doses of AF for 12 hours. Cells were then harvested and stained with annexin V-FITC/ propidium iodide (PI) apoptosis detection kit (Keygen Inc, Nanjing, China). Briefly, cells were gently trypsinized and then washed twice with PBS. The cells were resuspended in 500μl binding buffer and then 5μL of annexin V-FITC and 5μL of PI dyes were added. After incubation at room temperature for 10 minutes in the dark, samples were analyzed by a flow cytometer FC500 (Beckman Coulter Inc., Brea, CA, USA).

### Flow cytometric analysis of intracellular ROS production

ARPE-19 cells (1.0×10^5^/well) in 12-well culture plates were treated with different doses of AF for 12 hours. Cells were then incubated with 10μM DCFDA in serum free media at 37°C for 20 minutes to assess the level of ROS. Cells were washed once with PBS and were trypsinized. Cells were then washed one time with PBS, resuspended in 1m PBS, and were analyzed by the flow cytometer FC 500.

### BrdU incorporation assay in vitro

ARPE-19 cells were cultured on 0.1% gelatin-coated glass slides in 12-well plates for 12 hours. Cells were divided into a control group and three treatment groups for 24 hours: treatment with 1.0 μM AF, treatment with EGF (100ng/ml), and a combined treatment with EGF (100ng/ml) plus 1.0 μM AF. Then cells were incubated with 30μM BrdU for 4 hours, fixed with 4% PFA for 30 minutes at room temperature, washed with PBS three times, and acid-denatured with 2M hydrochloric acid in 0.1% phosphate buffer solution containing 0.1% Tween20 (PBST) for 30 minutes. Cells were further blocked with 5% bovine serum albumin for 1 hour following by a primary anti-BrdU antibody incubation for 2 hours at room temperature. Cells were then washed with PBS thrice and then were incubated with secondary antibody for 1 hour. After three rinses in PBS, the specimens were mounted onto glass slides by FluoroShield™ with DAPI(Sigma Aldrich, MO, USA) and were observed by using a confocal microscope(LSM 780,Carl Zeiss, Jena, Germany).

### Cell migration assay

ARPE-19 cells grew to confluence in 12-well plates. Then cells were divided into a control group and three treatment groups: treatment with 1.0 μM AF, treatment with EGF (100ng/ml), and a combined treatment with EGF (100ng/ml) plus 1.0 μM AF. All groups were subjected to “wound injury” assay with a 10 μl plastic pipette tip as in our previous study^18^. The ARPE-19 cells migration was determined by measuring wound areas in cell monolayers after 24 hours. Wound images were captured by an inverted microscope Nikon TE2000 (Nikon Instech Co., Tokyo, Japan). The distance of the wound healing (% closed) was measured and analyzed.

### Western blot analysis

All immunoblotting were performed as previously described [18]. In brief, after treated by reagents, ARPE-19 cells were washed twice by cold PBS and then were lysed in 1×SDS loading buffer. Protein samples were denatured in 100°C heat for 10 minutes, separated in 8~10% polyacrylamide gels, transferred to polyvinylidene difluoride membrane and blocked with 5% milk diluted in 0.1% PBST. Membranes were incubated with the specified primary antibodies as showed in Table 1 at 4°C over night, rinsed thrice in PBST, incubated with horseradish peroxidase-conjugated secondary antibodies (1:2000 in PBST) for 2 hours at room temperature. After three rinses in PBST, specific protein bands were developed by addition of chemiluminescence detection solution (Advansta Inc., Menlo Park, CA, USA) and then were visualized by Bio-Rad ChemiDoc XRS system (Bio-Rad, Hercules, CA, USA).The densities of various protein band were analyzed and β-actin was used as an internal control.

**Table 1:** Primary antibodies used for immunodetection.

Name	Species	Manufacturer	Product number	Application	Dilution
pEGFR	Rabbit	Cell Signaling	3777	WB, IF	1:1000(WB),1:100(IF)
EGFR	Rabbit	Cell Signaling	4267	WB, IF	1:1000(WB),1:100(IF)
pP38MAPK	Rabbit	Cell Signaling	4511	WB, IF	1:1000(WB),1:100(IF)
P38MAPK	Rabbit	Cell Signaling	9212	WB, IF	1:1000
pJNK	Rabbit	Cell Signaling	4668	WB	1:1000
pJNK	Mouse	Cell Signaling	9255	IF	1:100
JNK	Rabbit	Cell Signaling	9252	WB	1:1000
pERK	Rabbit	Cell Signaling	4370	WB, IF	1:1000(WB),1:100(IF)
ERK	Rabbit	Cell Signaling	9102	WB	1:1000
p-c-Jun	Rabbit	Cell Signaling	3270	WB, IF	1:1000(WB),1:100(IF)
c-Jun	Rabbit	Cell Signaling	9165	WB	1:1000
pMAPKAPK2	Rabbit	Cell Signaling	3007	WB, IF	1:1000(WB),1:100(IF)
MAPKAPK2	Rabbit	Cell Signaling	3042	WB	1:1000(WB),1:100(IF)
pHSP27	Rabbit	Cell Signaling	9709	WB, IF	1:1000(WB),1:100(IF)
HSP27	Mouse	Cell Signaling	2402	WB	1:1000 (WB)
BrdU	Mouse	Proteintech	66241	IF	1:500

WB, Western blot; IF, immunofluorescence.

### Immunofluorescence

ARPE-19 cells were cultured on 0.1% gelatin-coated glass coverslips in 12-well plates for 12 hours. Cells were treated by different reagents, fixed with 4% paraformaldehyde in PBS for 15 minutes at room temperature and then rinsed three times in PBS. Cells were permeabilized with 0.1% triton-X buffer for 3 minutes, rinsed thrice in PBS, blocked in 5% horse serum diluted in PBS for 1 hour. Cells were then incubated with specified primary antibodies as showed in Table 1 for 2 hours at room temperature, rinsed thrice in PBS, followed by incubation with Alexa Fluor^®^ 488- or 594- conjugated secondary antibodies for 1 hour. After three rinses in PBS, cells on coverslips were mounted onto glass slides by FluoroShield™ with DAPI (Sigma Aldrich, MO, USA) and were observed by using LSM 780 confocal microscope.

### Statistical analysis

Experiments were carried out at least in triplicate and values are expressed as mean± SEM. Difference between two groups was analyzed by Student’s t-test, and between three or more groups was analyzed by one-way ANOVA multiple comparisons followed by post-hoc Tukey test using GraphPad Prism 5 (GraphPad Software, LaJolla, CA, USA). Significance is indicated as follows: *P < 0.05, **P < 0.01, except where indicated otherwise.

## Results

### AF inhibited ARPE-19 cells survival in a dose and time-dependent manner

**Fig 1:**
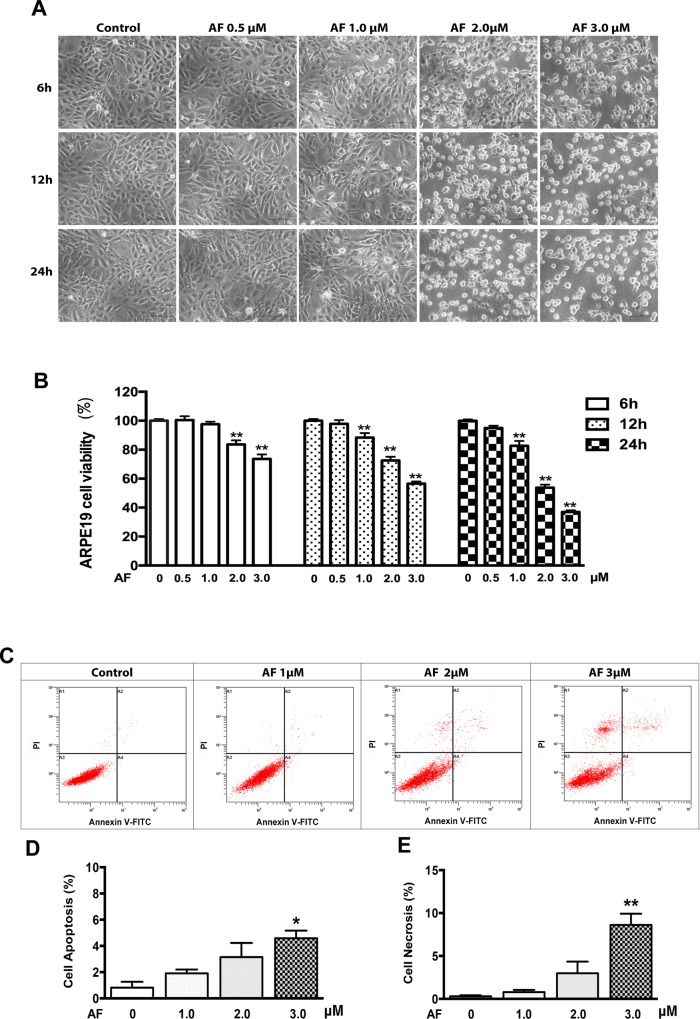
Auranofin inhibits ARPE-19 cells survival. (**A**) Phase-contrast photomicrographs of ARPE-19 cells after 0–3.0 μM AF treatment for 6, 12 and 24 hours. Scale bar = 50μm. (**B**) ARPE-19 cells were treated with AF at 0–3.0 μM for 6, 12 and 24 hours. Cells viability was measured by an MTT assay. The cell viability is expressed as a percentage of control and data are from three independent experiments, n = 4. (**C**) ARPE-19 cells were treated with AF at 1.0 μM, 2.0 μM and 3.0 μM for 12 hours and stained with PI and Annexin V-FITC. Cells death was detected by flow cytometery (in dot plots, necrosis is only PI^+^ in area 1(A1), late apoptosis is annexin V^+^+PI^+^ in area 2(A2), normal living is annexin V^−^+PI^−^ in area 3 (A3), early apoptosis is annexin V^+^ in area 4 (A4)). (**D**) Quantification of ARPE-19 cell apoptosis (early apoptosis: annexin V^+^, late apoptosis: annexin V^+^+PI^+^) induced by AF, n = 3. (**E**) Quantification of ARPE-19 cell necrosis (PI^+^) induced by AF, n = 3. All data are means ± SEM, *P<0.05, **P<0.01, compared with control.

In the present study, the results of the microscopic observation showed cell shrinkage and cell body rounding after AF treatment at or above dose levels of 2.0 μM (Fig 1A). The cell viability was measured by a MTT assay. As shown in Fig 1B, treatment with AF (0–3.0 μM) for 6, 12 and 24 hours caused significant inhibition of cell viability in a dose and time-dependent manner. To confirm the cytotoxicity of AF, cell death was also detected by flow cytometery (Fig 1C). As shown in Fig 1D and 1E, compared with control group (apoptosis, 0.8% ± 0.3%; necrosis, 0.3% ± 0.1%), treatment with 1.0 μM AF for 12 hours did not cause significant induction of cell apoptosis (1.9% ± 0.2%) and necrosis (0.8% ± 0.2%). Similarly, treatment with 2.0 μM AF for 12 hours did not cause significant induction of cell apoptosis (3.1% ± 1.1%) and necrosis (3.0% ±1.4%). Only treatment with the highest dose—3.0 μM AF for 12 hours, caused significant induction of cell apoptosis (4.6% ± 0.6%, *P*<0.05) and necrosis (8.6% ± 1.3%, *P*<0.05).

### AF induces an increase in ROS in ARPE-19 Cells

**Fig 2:**
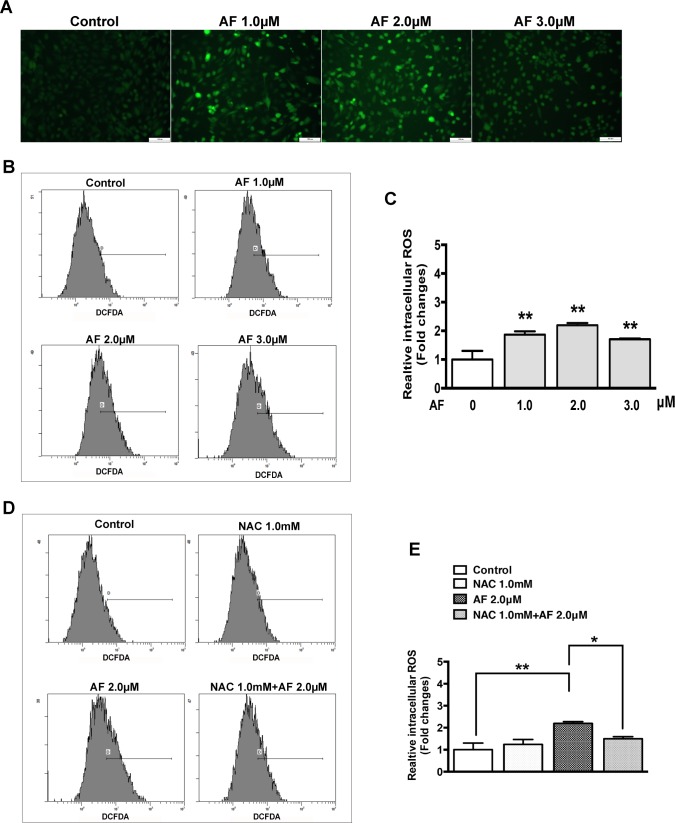
Auranofin induces an increase in reactive oxygen species (ROS) in ARPE-19 cells. (**A**) Fluorescent photomicrographs of ARPE-19 cells after treatment with 0–3.0 μM AF for 12 hours and DCFDA staining for 20 minutes. Green fluorescence of DCF was observed by fluorescence microscope. Scale bar = 100μm. (**B**) The fluorescence density of DCF was measured by flow cytometery after ARPE-19 cells were treated with 0–3.0 μM AF for 12 hours and stained with DCFDA for 20 minutes. (**C**) The histogram represents quantified data of fluorescence density of DCF shown in panel B (data are means ± SEM, n = 3, *P<0.05, **P<0.01, compared with control). (**D**) The fluorescence density of DCF was measured by flow cytometry after ARPE-19 cells with or without 1.0 mM N-acetylcysteine (NAC) pretreatment for 2 hours were treated by 2.0 μM AF for 12 hours and stained with DCFDA for 20 minutes. (**E**) The histogram represents quantified data of fluorescence density of DCF shown in panel D (data are means ± SEM, n = 3, *P<0.05, **P<0.01).

To elucidate some intracellular mechanisms in RPE cells after AF treatment, the intracellular ROS production was measured by using a non-fluorescent dye DCFDA. This non-fluorescent probe can be oxidized to the fluorescent product DCF by intracellular ROS production [19]. As shown in Fig 2A, a significant increase in the fluorescent product DCF was observed in ARPE-19 cells with all doses of AF treatment (1.0, 2.0, and 3.0μM). The level of fluorescent DCF which corresponds to the level of relative ROS production was detected by using flow cytometry (Fig 2B). The data showed that, compared to control, relative ROS production after application of AF increased significantly by 1.9, 2.2, and 1.7 times, respectively (Fig 2D). Pretreatment with antioxidant NAC (1.0 mM) significantly suppressed the increase of ROS production induced by 2.0 μM AF (Fig 2C and 2E).

### The effect of AF on EGFR/MAPKs signaling pathway in ARPE-19 cells

**Fig 3:**
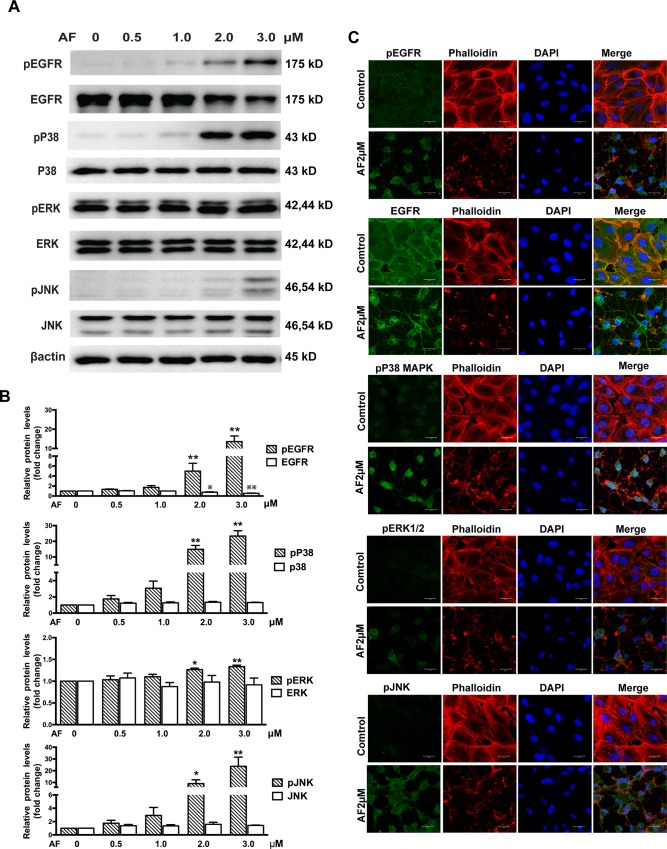
Auranofin induces phosphorylation of EGFR/MAPK. (**A**) ARPE-19 cells were treated with different doses of AF for 12 hours. Cell lysates were subjected to Western blot for total and phosphorylated EGFR, P38MAPK, ERK, JNK, with respective antibodies. β-actin was used as an internal control. (**B**) Quantitative data of Western blot presented in panel **A** from three independent experiments. The levels of phosphorylated protein were compared with the control levels, *P < 0.05,**P < 0.01; the levels of total protein were compared with control levels,^※^*P* < 0.05,^※※^*P* < 0.01. (**C**) Immunofluorescence microphotographs of total and phosphorylated EGFR (green), phosphorylated P38MAPK (green), phosphorylated ERK(green), phosphorylated JNK(green) with phalloidin (red) and DAPI (blue) after ARPE-19 cells were treated with 2.0 μM AF for 12 hours. Scale bar = 20μm.

**Fig 4:**
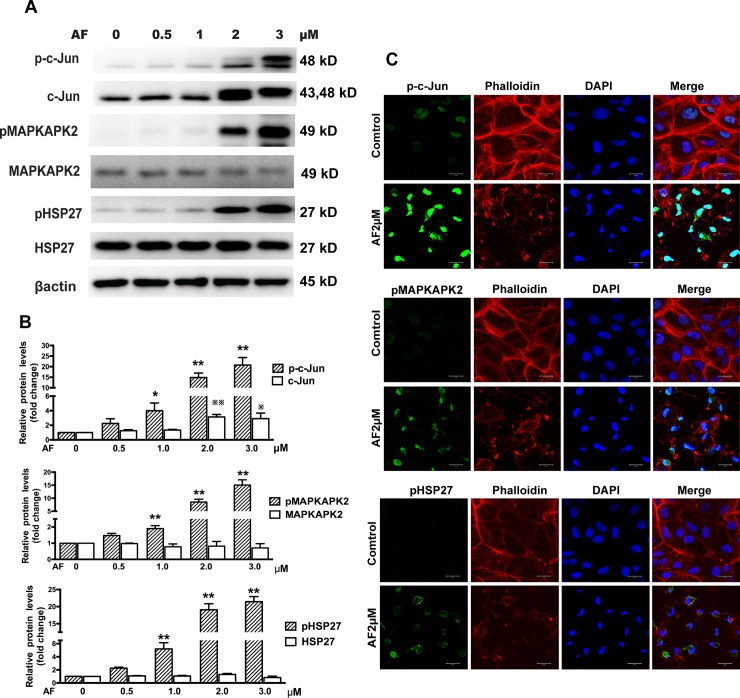
Auranofin induces phosphorylation of c-Jun, MAPKAPK2, and HSP27. (**A**) ARPE-19 cells were treated with different doses of AF for 12 hours. Cell lysates were subjected to Western blot for determination of total and phosphorylated c-Jun, MAPKAPK2, and HSP27 with respective antibodies. β-actin was used as a loading control. (**B**) Quantitative data of Western blot results shown in panel A from three independent experiments. Results are shown as mean±SEM. The levels of the phosphorylated protein were compared with the control, * *P* < 0.05, ** *P* < 0.01. The levels of the total protein were compared with the control, ^※^*P* < 0.05, ^※※^*P* < 0.01. (**C**) Immunofluorescence microphotographs of phosphorylated c-Jun(green), phosphorylated MAPKAPK2 (green), phosphorylated HSP27 (green) with phalloidin (red) and DAPI(blue) after ARPE-19 cells were treated with 2.0 μM AF for 12 hours. Scale bar = 20μm.

We next tested the effect of AF on EGFR/MAPK signaling pathways in ARPE-19 cells. Western blot results showed that treatment with doses above 1.0 μM of AF for 12 hours caused a significant upregulation of phosphorylated EGFR and down-regulation of total EGFR. We also observed that AF induced a significant increase in the phosphorylation of P38MAPK, ERK, and JNK, without affecting the expression levels of these proteins (Fig 3A and 3B). Furthermore, we detected the effect of AF on MAPKAPK2, HSP27, and c-Jun. We noticed that AF (≥ 2.0 μM) induced significant phosphorylation of MAPKAPK2, HSP27 and c-Jun. However, AF had different effects on the expression levels of total MAPKAPK2, HSP27 and c-Jun. We found that the expression level of total c-Jun protein was up-regulated, whereas the expression levels of total MAPKAPK2 protein was slightly reduced after application of 2.0μM and 3.0μM AF, and expression levels of total HSP27 protein were slightly increased after application of 2.0 μM AF, but reduced after 3.0μM AF (Fig 4A and 4B).

Immunofluorescence microscopic evaluation of ARPE-19 cells showed that treatment with AF (2.0 μM) induced translocation of EGFR from the cell membrane to the cytoplasm and significantly increased the levels of phosphorylated EGFR, JNK, ERK, P38MAPK, c-Jun, MAPKAPK2, and HSP27. The F-actin staining with a fluorescent phalloidin probe revealed cell body shrinkage, cytoskeleton destruction, and F-actin fibers destabilization in ARPE-19 cells. Moreover, nuclear shrinkage was also observed with DAPI staining. (Figs 3C and 4C).

### AF inhibits EGF-induced proliferation and migration of ARPE-19 cells

**Fig 5:**
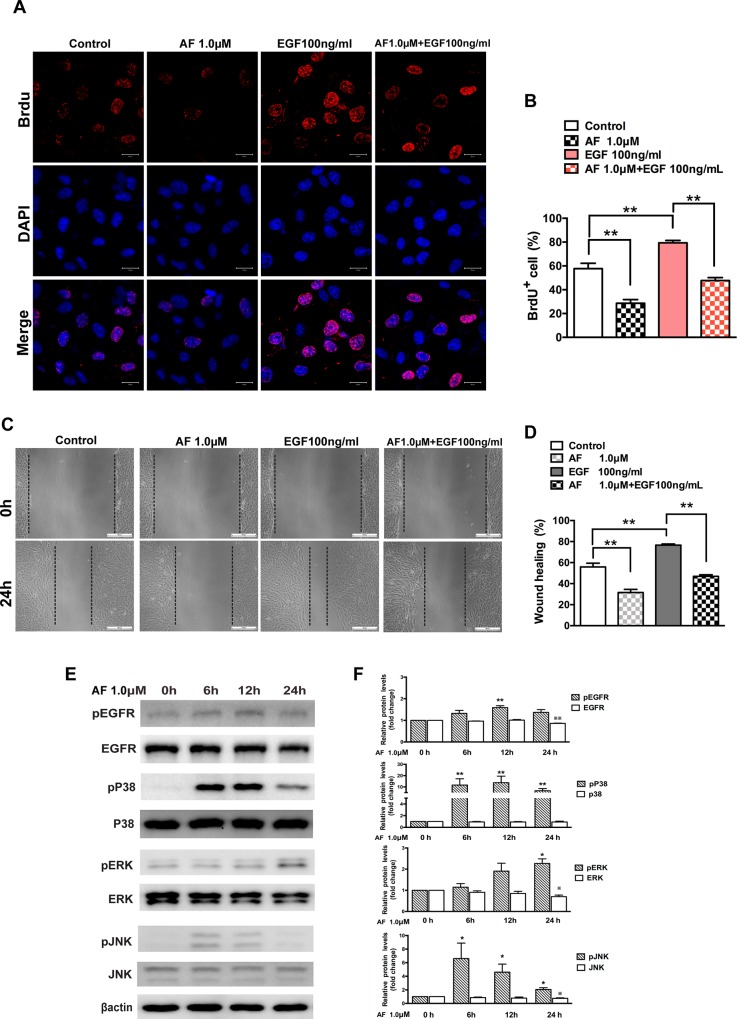
Auranofin inhibits EGF-dependent proliferation and migration of ARPE-19 cells. (**A**) Immunofluorescence microphotographs of proliferating ARPE-19 cell with BrdU (red) and DAPI (blue) staining after ARPE-19 cells were untreated or treated with AF (1.0 μM) in the absence or presence of EGF (100ng/ml) for 24 hours and then subjected to BrdU labeling for 4 hours, followed by immunostaining with anti-BrdU antibody and DAPI. Scale bar = 20μm. (**B**) Quantitation data of the number of BrdU^+^ cells shown in panel A. (**C**) ARPE-19 cells were subjected to wound healing assay, and then were left untreated or treated with AF (1.0 μM) in the absence or presence of EGF (100 ng/ml) for 24 hours. Scale bar = 100μm. (**D**) Quantitation of the results shown in panel C. (E) ARPE-19 cells were treated with 1.0 μM AF for 6, 12 and 24 hours. Cell lysates were subjected to Western blot for determination of total and phosphorylated EGFR, P38MAPK, ERK and JNK proteins. β-actin was used as a loading control. (**F**) Quantitative data of Western blot results shown in panel E from three experiments. The levels of the phosphorylated protein were compared with the control, * *P* < 0.05, ** *P* < 0.01. The levels of the total protein were compared with the control, ^※^*P* < 0.05, ^※※^*P* < 0.01. All data are mean ± SEM.

The effect of AF on ARPE-19 cells proliferation was detected by a BrdU incorporation assay. The number of BrdU+ ARPE-19 cells after treatment with AF (1.0 μM) group was reduced on average by 29% compared to control, while in the EGF treatment group, the number of BrdU+ ARPE-19 cell increased on average by 22%. Furthermore, the number of BrdU+ cells in the combined treatment group (EGF+AF) was reduced on average by 32%, compared to the EGF treatment group (Fig 5A and 5B).

In the cell migration assay, we observed the healing of monolayer cell after ARPE-19 cell subjected to “wound injury” and we found that wound healing in the AF-treatment group was suppressed on average by 24.4% compared to the control when cells were treated with AF for 24 hours. In contrast, wound healing in the EGF-treatment group was enhanced on average by 20.7% compared to control. Notably, treatment with AF significantly inhibited EGF-stimulated wound healing on average by 29.6% (Fig 5C and 5D). To demonstrate the mechanism of lower does of AF on RPE cell, we tested the effect of treatment with 1.0 μM AF for 6, 12 and 24 hours on EGFR/MAPK proteins. As Fig 5E and 5F show, treatment with 1.0μM of AF for 6, 12 and 24 hours induced phosphorylation of EGFR, P38MAPK, ERK and JNK proteins. Treatment with 1.0 μM of AF for 6 and12 hours did not affect the expression levels of total EGFR, P38MAPK, ERK and JNK protein. However, treatment with 1.0 μM of AF for 24hours caused noticeable reduction of total EGFR (15%), ERK (30%) and JNK (25%) proteins, except for P38MAPK protein.

### AF interferes with EGFR/MAPK signaling induced by EGF in ARPE-19 cells

**Fig 6:**
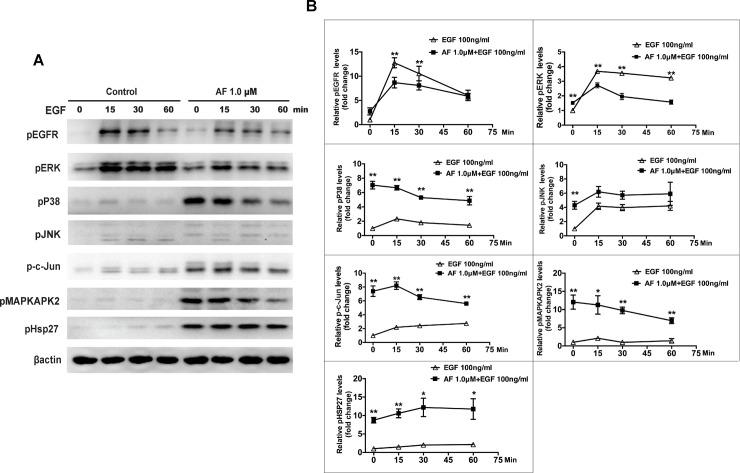
Auranofin interferes with EGF-induced EGFR/MAPK signaling in ARPE-19 cells. (**A**) ARPE-19 cells were pretreated with AF (1.0 μM) in serum free media for 12 hours, and then stimulated by 100ng/ml EGF for 0, 15, 30, and 60 minutes. Cell lysates were subjected to Western blot for phosphorylated EGFR, P38MAPK, ERK, JNK, c-Jun, MAPKAPK2, and HSP27 with respective antibodies. β-actin was used as a loading control. (**B**) Quantitation of Western blot results shown in panel **A** from three independent experiments. Comparison between the control group and AF pretreatment group at the same time point. * *P*<0.05, ** *P*<0.01.

To investigate the molecular mechanism of AF inhibiting EGF-dependent cell proliferation and migration, we probed the effect of AF on EGF/EGFR/MAPK signaling. EGF induced significant phosphorylation of EGFR and ERK, and slight phosphorylation of P38MAPK, JNK, MAPKAPK2, HSP27, and c-Jun. By contrast, treatment with 1.0 μM AF for 12 hours caused slight phosphorylation of EGFR and ERK, but significant phosphorylation of P38MAPK, JNK, MAPKAPK2, c-Jun, and HSP27. The phosphorylation of EGFR and ERK, that was induced by EGF treatment for 15 and 30 minutes, was significantly inhibited by pretreatment with 1.0 μM of AF for 12 hours (Fig 6A and 6B).

### AF-induced EGFR/MAPK signaling is suppressed by NAC

**Fig 7:**
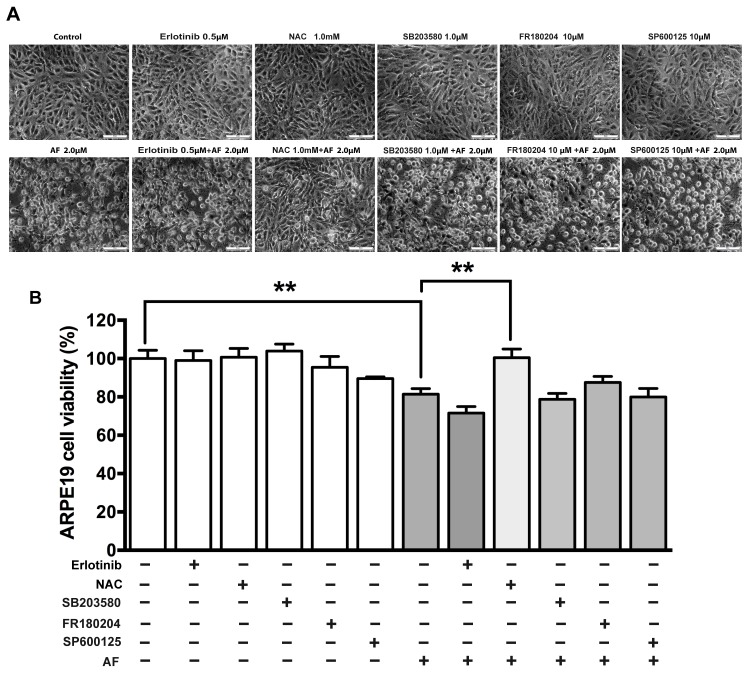
The NAC rescued AF-induced inhibition of ARPE-19 cell viability. ARPE-19 cells were treated by 2.0 μM AF combined with or without 0.5 μM erlotinib, 1.0 mM NAC, 1.0 μM SB203580, 10 μM FR180204 and 10 μM SP600125 treatment for 12 hours. (**A**) Phase-contrast photomicrographs of ARPE-19 cells after treatment. Scale bar = 50μm. (**B**) Cells viability of ARPE-19 cells after treatment was measured by an MTT assay. The cell viability is expressed as a percentage of control and data are from three independent experiments. All data are means ± SEM, n = 4, **P<0.01.

**Fig 8:**
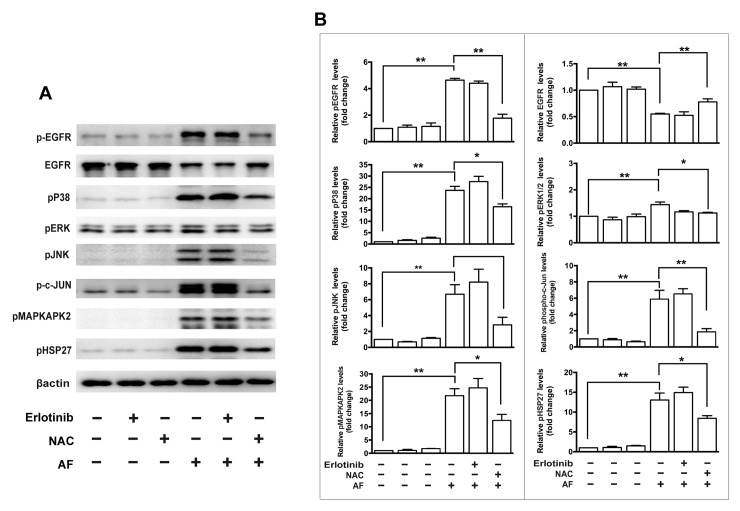
The effect of erlotinib and N-acetylcysteine (NAC) on auranofin-induced EGFR/MAPK signaling in ARPE-19 cells. (**A**) ARPE-19 cells were pretreated with 0.5μM erlotinib or 1.0 mM NAC for 2 hours and further treated with AF for another 12 hours. Cell lysates were subjected to Western blot analysis. Total and phosphorylated protein level of EGFR, phosphorylated P38MAPK, phosphorylated ERK, phosphorylated JNK, phosphorylated c-Jun, phosphorylated MAPKAPK2, and phosphorylated HSP27 were detected. β-actin was used as a loading control. (**B**) Quantitation analysis of the results presented in panel **A** from three independent experiments. * *P* < 0.05, ** *P* < 0.01.

**Fig 9:**
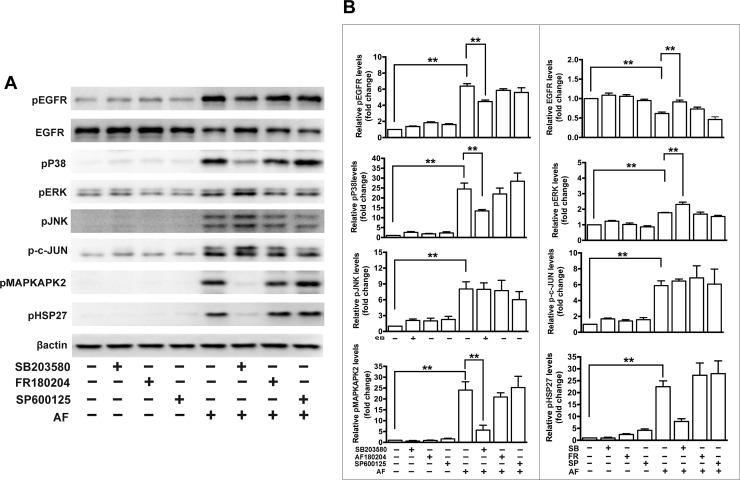
The effect of MAPK inhibitors on auranofin-induced EGFR/MAPK signaling in ARPE-19 cells. (**A**) ARPE-19 cells were pretreated with 1.0 μM SB203580 (P38MAPK inhibitor), 10 μM FR180204 (ERK inhibitor) or 10 μM SP600125 (JNK inhibitor) for 2 hours and further treated with 2.0 μM AF for another 12 hours. Cell lysates were subjected to Western blot analysis. Total and phosphorylated protein level of EGFR, phosphorylated P38MAPK, phosphorylated ERK, phosphorylated JNK, phosphorylated c-Jun, phosphorylated MAPKAPK2 and phosphorylated HSP27 were detected. β-actin was used as a loading control. (**B**) Quantitation analysis of the results presented in panel **A** from three independent experiments. * *P* < 0.05, ** *P* < 0.01.

In addition, we tested effects of the antioxidant NAC and several inhibitors of EGFR/MAPK signaling on cell viability and the AF-induced signaling in ARPE-19 cells. Specifically, we applied an inhibitor of EGFR (erlotinib), an inhibitor of P38MAPK (SB203580), an inhibitor of ERK (FR180204) and an inhibitor of JNK (SP600125). The cell viability assay showed that NAC significantly rescued AF-induced inhibition of ARPE-19 cell viability, but inhibitors including erlotinib, SB203580, FR180204 and SP600125 did not affect inhibition of cell viability induced by AF (Fig 7). Western blot results showed that pretreatment with 1.0 μM NAC for 2 hours significantly blocked the decrease of total EGFR and the increase of phosphorylated EGFR, JNK, ERK, P38MAPK, c-Jun, MAPKAPK2, and HSP27 induced by treatment with 2.0 μM AF for 12 hours (Fig 8). In addition, the application of 1.0 μM SB203580 for 2 hours significantly blocked 2.0 μM AF-induced the reduction of total EGFR and the increase of phosphorylated EGFR, P38MAPK, MAPKAPK2, and HSP27 (Fig 9). However, pretreatment with 0.5 μM erlotinib, 10 μM FR180204 and 10 μM SP600125 for 2 hours had no significant effect on 2.0 μM AF- induced EGFR/MAPK signaling.

## Discussion

AF is a drug originally developed in the 1970s for the treatment of rheumatoid arthritis [12]. Because of its potent anti-inflammatory, anti-proliferative and other properties, AF was also tested and has shown potential in a broad range of therapeutic indications for the treatment of different diseases including cancers, neurodegenerative disorders and infection diseases [13]. Based on this information and our previous experience with this drug [18], we decided to test if AF affects RPE cell proliferation to evaluate its potential as treatment for PVR, a disease associated with RPE cell proliferation [2].

Our results clearly demonstrated that AF exhibited anti-proliferative effects on RPE cells in vitro in the low micromolar range. Furthermore, AF showed numerous and varied effects on important parts of the EGFR signaling pathways consistent with its anti-proliferative action. Historically, the main mechanism of AF action was inhibiting the thioredoxin reductases [20]. Thioredoxin reductases are involved in the reduction of thioredoxins, small proteins important in the reduction of ROS [21] and in cell-to-cell communication [22]. A recent study showed that AF induced cell apoptosis through ROS-mediated endoplasmic reticulum stress and mitochondrial dysfunction [23]. In our previous study, we found that AF could cause reduction of TrxR2 and Trx2 proteins in endothelial cells [18]. In the present study, our results showed that AF inhibited RPE cells viability and induced RPE cell death by increasing ROS production. Pretreatment with antioxidant NAC significantly blocked AF-induced ROS increase.

EGFR plays an important role in initiating RPE cell migration and proliferation [7]. The MAPK pathway, mainly including ERK, JNK, and P38MAPK, is important downstream signaling of EGFR signaling pathway and is involved in a wide range of cellular responses such as differentiation [24], proliferation and migration [25,26], and apoptosis [27,28] in RPE cells. A recent study showed that various stresses also trigger significant pathways of ligand-independent EGFR trafficking and signaling [29]. Filosto et al. have found that oxidative stress caused abnormal phosphorylation and activated conformation of EGFR [30]. Some data suggested that the ERK activation mediated by EGFR plays an active role in oxidative stress-induced apoptosis of cells [31] and oxidative stress-dependent activation of JNK and P38MAPK is also involved in cell apoptosis [32,33]. Froscio et al. have demonstrated that AF enhanced the phosphorylation of EGFR in the epidermoid carcinoma cell lines [15]. Some studies also showed that AF caused phosphorylation of P38MAPK, ERK, JNK, and MAPKAPK2 [16,17], however, whether similar processes occur in RPE cells is not clear. Our present results confirm and expand upon these earlier findings in the context of cultured human RPE cells. Moreover, our data show that AF significantly reduced the expression of total EGFR and caused phosphorylation of many nodes of EGFR/MAPK signaling pathway, including EGFR, P38MAPK, JNK, ERK, c-Jun, MAPKAPK2, and HSP27 (Figs 3 and 4). MAPKAPK2 is a direct target of P38MAPK and HSP27 was identified as the major substrate of MAPKAPK2, which are known to transduce a range of extracellular signals that result in cell division and differentiation, apoptosis, and change in cell motility [34]. Furthermore, c-Jun is a component of the transcription factor activator protein-1 and is regulated by JNK, which regulated cell cycle progression and apoptosis by distinct mechanisms in fibroblasts [35]. To the best of our knowledge, this is the first study to show that AF induced phosphorylation of HSP27 and c-Jun. Moreover, our data show that HSP27 protein appears to a slight increase at 2.0 μM of AF and reduction at 3.0 μM of AF (Fig 4A), which could suggest a compensatory mechanism for the production of the HSP27 protein in this setting. Overall, these results support our initial hypothesis that AF can suppress RPE cells survival through activating multiple targets in EGFR/MAPK signaling pathway.

Several studies have shown that EGF induces the EGFR/MAPK signal transduction pathway and contributes to the survival, proliferation and migration of RPE cells [6–8]. Therefore, EGF is considered a major factor in the development of PVR [8,11]. In this study, we found that EGF-dependent proliferation and migration in RPE cell were inhibited by 1.0 μM AF (Fig 5). Interestingly, pretreatment with AF significantly inhibited EGF-induced phosphorylation of EGFR and ERK (Fig 6). These results provide an additional support to the hypothesis that AF could be used as a treatment agent for PVR by clarifying an additional mechanism of action, namely AF-induced inhibition of proliferation and migration of RPE cells through affecting EGF/EGFR/MAPK signaling pathway.

It has been reported that ROS scavenger NAC inhibited phosphorylation of P38MAPK and activation of caspases induced by AF [16]. A recent study showed that the thioredoxin-mimetic peptides selectively prevented AF-mediated phosphorylation of JNK and P38MAPK [17]. In this study, we also found that NAC significantly rescued AF-induced inhibition of ARPE-19 cells viability and blocked AF-induced down-regulation of total EGFR and up-regulation of phosphorylated EGFR, ERK, P38MAPK, JNK, MAPKAPK2, c-Jun, and HSP27. In addition, we found the P38MAPK inhibitor (SB203580), but not inhibitors of EGFR, ERK and JNK, significantly blocked down-regulation of total EGFR and blocked AF-induced phosphorylation of EGFR, P38MAPK, MAPKAPK2 and HSP27. These data suggested that the AF-induced ROS production might be a trigger of a series of phosphorylation of EGFR/MAPK signaling, and the P38MAPK might be an important target for the AF-induced phosphorylation of EGFR/MAPK signaling pathway.

Several previous studies showed that AF inhibits cell survival in different types of cells, such as the survival of endothelial cells [18,36], neutrophil cells [37], and cancer cells [38]. On the other hand, some authors found that AF can protect neurons by inhibiting astrocyte-induced neuronal cell death, and by direct neuroprotection [39]. The finding that AF (1.0 μM) shows effects on proliferating RPE cells supports the possibility that this drug could be a relatively selective inhibitor of RPE cell proliferation in PVR. This is further supported by the fact that AF administration in clinical settings is not typically associated with ocular adverse effects [40]. However, further studies in vivo are required to establish whether AF inhibits proliferation of RPE, without toxic effects on neural retina.

In conclusion, the results of our study show that AF can suppress the survival of proliferating RPE cells through ROS-dependent phosphorylation of EGFR/MAPK signaling pathway, and thus is hopeful to treatment of abnormal survival of RPE cells in PVR.
