# Correction: Correction: Auranofin Inhibits Retinal Pigment Epithelium Cell Survival through Reactive Oxygen Species-Dependent Epidermal Growth Factor Receptor/ Mitogen-Activated Protein Kinase Signaling Pathway

**DOI:** 10.1371/journal.pone.0187417

**Published:** 2017-10-27

**Authors:** The PLOS ONE Staff

## Notice of Republication

This correction was republished on March 9, 2017, to correct an error in Fig 5 within the correction article. The publisher apologizes for the error. Please download this article again to view the correct version. The originally published, uncorrected article and the republished, corrected article are provided here for reference.

## Supporting information

S1 File Originally published, uncorrected article. (PDF)

S2 File Republished, corrected article. (PDF)
