# Supplementary material for: Correction: Correction: Auranofin Inhibits Retinal Pigment Epithelium Cell Survival through Reactive Oxygen Species-Dependent Epidermal Growth Factor Receptor/ Mitogen-Activated Protein Kinase Signaling Pathway
Source: PLoS One. 2017 Oct 27;12(10):e0187417. doi: 10.1371/journal.pone.0187417 (PMC5659781; doi:10.1371/journal.pone.0187417)
Supplement: S1 File — (PDF) [file pone.0187417.s001.pdf]

PLOS ONE

CORRECTION

Correction: Auranofin Inhibits Retinal Pigment Epithelium Cell Survival through Reactive Oxygen Species-Dependent Epidermal Growth Factor Receptor/ Mitogen-Activated Protein Kinase Signaling Pathway

Xiaodong Chen, Radouil Tzekov, Mingyang Su, Haiyan Hong, Wang Min, Aidong Han, Wensheng Li

There are a number of errors in Table 1. The "Application" value for "P38MAPK" in line 4 should read "WB". The "Dilution" value for "MAPKAPK2" in line 13 should read "1:1000 (WB)". The "Dilution" value for "HSP27" in line 15 should read "1:1000". Please view the correct Table 1 here.

Check for updates

OPEN ACCESS

Citation: Chen X, Tzekov R, Su M, Hong H, Min W, Han A, et al. (2017) Correction: Auranofin Inhibits Retinal Pigment Epithelium Cell Survival through Reactive Oxygen Species-Dependent Epidermal Growth Factor Receptor/ Mitogen-Activated Protein Kinase Signaling Pathway. PLoS ONE 12(2): e0172599. doi:10.1371/journal.pone.0172599

Published: February 21, 2017

Copyright: © 2017 Chen et al. This is an open access article distributed under the terms of the Creative Commons Attribution License, which permits unrestricted use, distribution, and reproduction in any medium, provided the original author and source are credited.

Table 1. Primary antibodies used for immunodetection.

Name	Species	Manufacturer	Product number	Application	Dilution
pEGFR	Rabbit	Cell Signaling	3777	WB, IF	1:1000(WB),1:100(IF)
EGFR	Rabbit	Cell Signaling	4267	WB, IF	1:1000(WB),1:100(IF)
pP38MAPK	Rabbit	Cell Signaling	4511	WB, IF	1:1000(WB),1:100(IF)
P38MAPK	Rabbit	Cell Signaling	9212	WB	1:1000
pJNK	Rabbit	Cell Signaling	4668	WB	1:1000
pJNK	Mouse	Cell Signaling	9255	IF	1:100
JNK	Rabbit	Cell Signaling	9252	WB	1:1000
pERK	Rabbit	Cell Signaling	4370	WB, IF	1:1000(WB),1:100(IF)
ERK	Rabbit	Cell Signaling	9102	WB	1:1000
p-c-Jun	Rabbit	Cell Signaling	3270	WB, IF	1:1000(WB),1:100(IF)
c-Jun	Rabbit	Cell Signaling	9165	WB	1:1000
pMAPKAPK2	Rabbit	Cell Signaling	3007	WB, IF	1:1000(WB),1:100(IF)
MAPKAPK2	Rabbit	Cell Signaling	3042	WB	1:1000
pHSP27	Rabbit	Cell Signaling	9709	WB, IF	1:1000(WB),1:100(IF)
HSP27	Mouse	Cell Signaling	2402	WB	1:1000
BrdU	Mouse	Proteintech	66241	IF	1:500

WB, Western blot; IF, immunofluorescence.

doi:10.1371/journal.pone.0172599.t001

In Fig 5, Fig 5C shows the incorrect image under the "AF 1.0 μM" header for "0h". Please view the correct Fig 5 here.

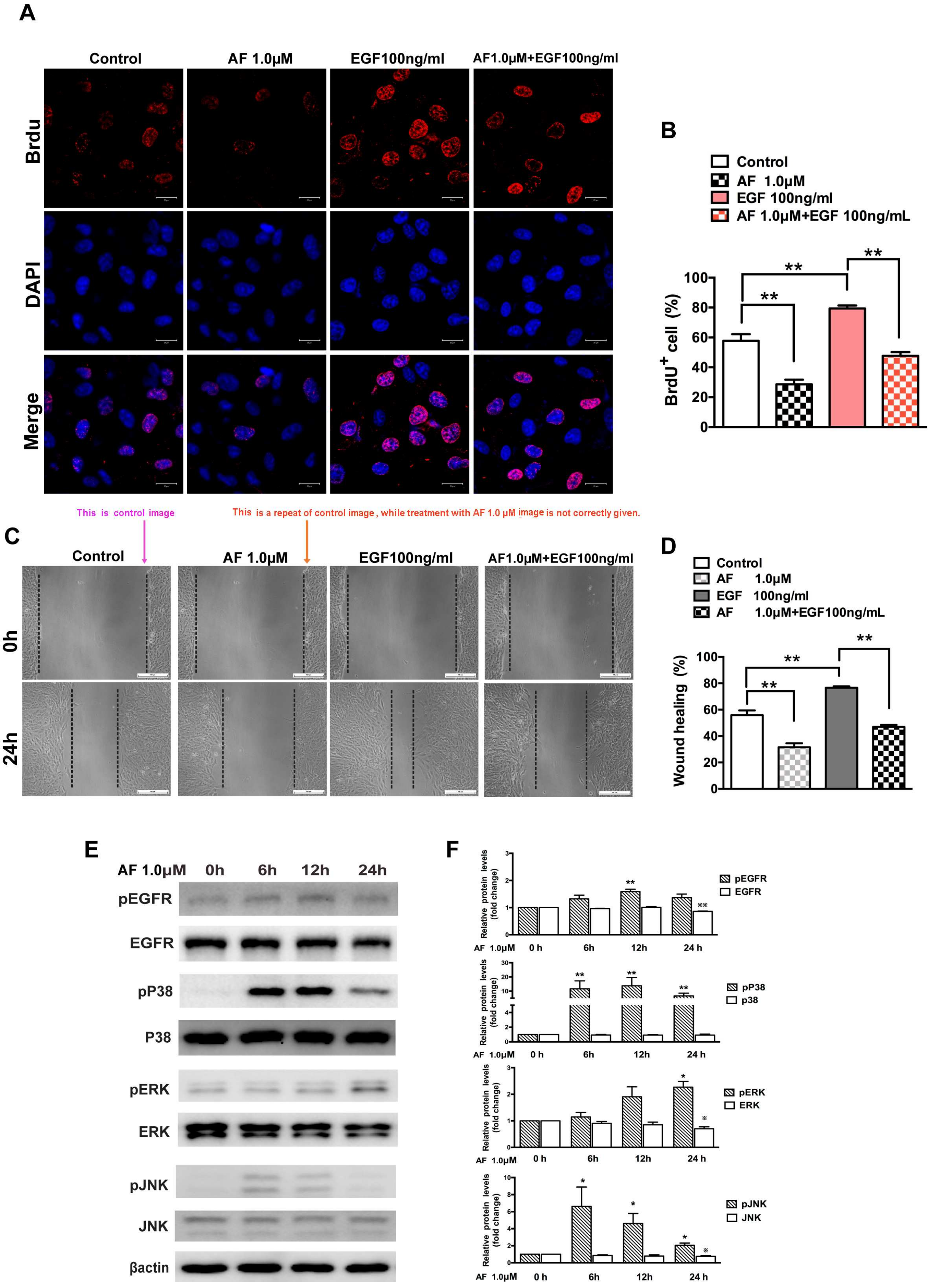
A
Control
AF 1.0μM
EGF100ng/ml
AF1.0μM+EGF100ng/ml
Brdu
DAPI
Merge
B
Control
AF 1.0μM
EGF 100ng/ml
AF 1.0μM+EGF 100ng/mL
BrdU+ cell (%)
This is control image
This is a repeat of control image , while treatment with AF 1.0 μM image is not correctly given.
C
0h
24h
D
Wound healing (%)
E
AF 1.0μM 0h 6h 12h 24h
pEGFR
EGFR
pP38
P38
pERK
ERK
pJNK
JNK
βactin
F
Relative protein levels (fold change)
pEGFR
EGFR
pP38
p38
pERK
ERK
pJNK
JNK
AF 1.0μM 0 h 6h 12h 24 h

Fig 5. Auranofin inhibits EGF-dependent proliferation and migration of ARPE-19 cells. (A) Immunofluorescence microphotographs of proliferating ARPE-19 cell with BrdU (red) and DAPI (blue) staining after ARPE-19 cells were untreated or treated with AF (1.0 μM) in the absence or presence of EGF (100ng/ml) for 24 hours and then subjected to BrdU labeling for 4 hours, followed by immunostaining with anti-BrdU antibody and DAPI. Scale bar = 20μm. (B) Quantitation data of the number of $BrdU^{+}$ cells shown in panel A. (C) ARPE-19 cells were subjected to wound healing assay, and then were left untreated or treated with AF (1.0 μM) in the absence or presence of EGF (100 ng/ml) for 24 hours. Scale bar = 100μm. (D) Quantitation of the results shown in panel C. (E) ARPE-19 cells were treated with 1.0 μM AF for 6, 12 and 24 hours. Cell lysates were subjected to Western blot for determination of total and phosphorylated EGFR, P38MAPK, ERK and JNK proteins. β-actin was used as a loading control. (F) Quantitative data of Western blot results shown in panel E from three experiments. The levels of the phosphorylated protein were compared with the control, * $P < 0.05$, ** $P < 0.01$. The levels of the total protein were compared with the control, ※$P < 0.05$, ※※$P < 0.01$. All data are mean ± SEM.

doi:10.1371/journal.pone.0172599.g001

Reference

1. Chen X, Tzekov R, Su M, Hong H, Min W, Han A, et al. (2016) Auranofin Inhibits Retinal Pigment Epithelium Cell Survival through Reactive Oxygen Species-Dependent Epidermal Growth Factor Receptor/Mitogen-Activated Protein Kinase Signaling Pathway. PLoS ONE 11(11): e0166386. doi:10.1371/journal.pone.0166386 PMID: 27846303
